# An investigation into the spiritual needs of neuro-oncology patients from a nurse perspective

**DOI:** 10.1186/1472-6955-12-2

**Published:** 2013-02-01

**Authors:** Aline Victoria Nixon, Aru Narayanasamy, Vivian Penny

**Affiliations:** 1 Research Nurse, Faculty of Medicine & Health Sciences, School of Biomedical Sciences, Queens Medical Centre, University of Nottingham, Nottingham, UK; 2 Associate Professor and Director of Ethnicity, Diversity and Spirituality (EDS) Hub, Faculty of Medicine & Health Sciences, School of Nursing, Midwifery & Physiotherapy, Queens Medical Centre, University of Nottingham, Nottingham, UK; 3 Associate Professor and Academic Lead for Adult Nursing, Faculty of Medicine & Health Sciences, School of Nursing, Midwifery & Physiotherapy, Queens Medical Centre, University of Nottingham, Nottingham, UK

**Keywords:** Spirituality, Spiritual care, Neuro-oncology, Critical incident techniques

## Abstract

**Background:**

Spiritual needs of cancer patients should be assessed and discussed by healthcare professionals. Neurosurgical nurses need to be able to assess and support neuro-oncology patients with their spiritual needs from diagnosis and throughout their hospital stay.

**Methods:**

Data were collected through questionnaires using a Critical Incident Technique (CIT) from neurosurgical nurses, findings were analysed using thematic analysis.

**Results:**

Nurses reported some awareness of their patients’ spiritual needs during their stay on neurosurgical units although some used expressions approximating what could be described as spiritual needs. Patients’ spiritual needs were identified as: need to talk about spiritual concerns, showing sensitivity to patients’ emotions, responding to religious needs; and relatives’ spiritual needs included: supporting them with end of life decisions, supporting them when feeling being lost and unbalanced, encouraging exploration of meaning of life, and providing space, time and privacy to talk. Participants appeared largely to be in tune with their patients’ spiritual needs and reported that they recognised effective strategies to meet their patients’ and relatives’ spiritual needs. However, the findings also suggest that they don’t always feel prepared to offer spiritual support for neuro-oncology patients.

**Conclusions:**

There is a need for healthcare professionals to provide spiritual care for neuro-oncology patients and their relatives. Although strategies were identified that nurses can use to support patients with spiritual needs further research is required to explore how effective nurses are at delivering spiritual care and if nurses are the most appropriate professionals to support neuro-oncology patients with spiritual care.

## Background

In cancer and palliative care the focus on spiritual care continues to grow. Policy, research and practical guidelines for health care professionals now routinely suggest that spiritual needs are an essential component of holistic health care assessment [1-3]. There is a body of evidence in nursing and health care literature to suggest that spirituality is integral to patients’ well-being [4,5]. The holistic approach to care, including cancer nursing is based on the premise that caring for the whole person constitutes the body, mind and spirit [6].

Although the focus of our research was on neuro-cancer nursing and spirituality, we draw from the general sources on spirituality and specialised literature on spirituality and cancer nursing because research is deplete in the area of our investigation, i.e., spirituality and neuro-cancer nursing. However, the literature on the general aspects of spirituality and spirituality and cancer nursing offers some directions on spirituality in nursing, hence our attention to such body of evidence to explicate current understanding to contextualise our research. Spirituality presents a problem for the nurse researcher as it is a complex and diverse phenomenon with no single authoritative definition [7]. Scheurich [7] suggests that this could be resolved by replacing the term ‘spirituality’ with ‘meanings and values of life’. Since spirituality is identified as a significant dimension for the holistic well-being of individuals [4,5], it is therefore important for nurses to recognise and respond to their patients’ spiritual needs. For the purpose of our study spirituality is seen to be a universal human phenomenon present in all individuals whether they are religious or not [1] and it is the essence of our being [8,9]. It gives meaning and purpose in our lives [10]. It is a dimension that comes into focus when an individual faces emotional stress, physical illness and death [10]. The diagnosis of neuro-cancer is a critical juncture in a person’s life and it is well documented in the literature that spirituality may offer strength, hope and comfort [11-13]. Several studies offer clinical evidence that patients use their spirituality as coping mechanisms when faced with critical junctures such as cancer and other life threatening illnesses [3,4,810,13].

‘Spiritual needs’ and ‘spirituality’ are concerned with the ‘spirit’ aspect of the human condition. Narayanasamy [8] and Puchalski [5] advance that ‘being spiritual’ decreases fear of death, increases comfort, and supports a positive perspective of death in ill patients. Patients’ may benefit from interventions that are sensitive, supportive, and responsive to their spiritual needs [11-13] as such it is important that we examine spiritual healthcare further. Oncology patients are likely to reflect on spiritual and existential issues due to the uncertainty of their future [14]. One group of cancer patients who have potential needs in this care area are those with a diagnosis of brain cancer, the focus of our research. Brain cancer threatens life itself but it is further complicated by a threat to the individuals personality and potential ‘loss of self’ due to the functional and cognitive deficits that accompany this disease [15-17].

There is confusion amongst healthcare professionals over who should be responsible for providing spiritual healthcare. Although Swinton [9] argues that all health professionals should be responsive to patient’s spiritual needs, a number of nursing scholars identify spiritual healthcare as a nursing role [8,11]. Nurses as healthcare professionals in the multidisciplinary team are best placed to attend to the spiritual needs of patients due to the nature of the relationship and the constant contact they have with patients. However, where good health care provisions for spiritual care by Hospital Chaplains and designated spiritual advisers exist, our stance is that nurses can work collaboratively with them to meet the holistic needs of patients, including spiritual needs. In the United Kingdom (UK), the Nursing and Midwifery Council (NMC) require that nurses are able to assess and manage the spiritual needs of their patients [18]. However, it has been reported that nurses are unclear about their role in spiritual care [19] and that this is a neglected area of nursing practice [20]. However, Thoresen and Harris [4] are positive that attitudes are changing with more medical and nursing schools including courses in spirituality within their curricula. In response to this, the Royal College of Nursing has produced guidelines and on-line resources on spirituality for nurses following a national survey of nurses’ perception of spirituality and spiritual care [2,21,22].

It is important that spirituality is addressed in health care as current evidence indicates that patients do have spiritual needs [1,4,11,23-26] and government policies [27,28] recommend that healthcare professionals should be addressing spiritual needs.

A stronger evidence is required to act upon the UK government’s policies, otherwise patients’ spiritual needs may remain unmet. There is some suggestion in the literature that nurses working on intensive wards are less likely to notice the spiritual needs of their patients than those who work with terminally ill cancer patients [14] and it is therefore important to understand how spiritual care is managed in the acute neurosurgical setting. This study sets out to highlight how nurses address the spiritual needs of neuro-cancer patients.

Although there is proliferating research interests into the spiritual needs of cancer patients in general the evidence is patchy about how nurses respond and manage spiritual care interventions for neuro-oncology patients. One research team attempted to investigate spirituality in relation to neuro-oncology patients but their study’s findings could be not generalised because it was based on small samples in Sweden [3,17,29]. However, the results from this study were indicative that neuro-oncology patients have spiritual needs and that strategies such as psychological and spiritual support can be used to enable them to cope with the distresses. On the basis of the above exploratory study, Strangy and Strang [17] proceeded to conduct a more focussed study of twenty patients with brain tumours in which they mapped strategies that patients used to cope with and apply meaning to their situation. Such coping strategies were described as information seeking, rationalisation, positive reappraisal of life and redefinition, believing in one’s own strengths, hope, belief in a cure, humour, distancing, control, bargaining, support from hospital staff, relationships with children, family, friends, work, music, hobbies, and faith. In this regard, Baldacchino, et al. [11] draw attention to the helpfulness of spiritual coping strategies in their study of clients receiving Hospice Care.

Furthermore, from interviews with nurses Strang, Strang, and Ternestedt [29] found that nurses’ opinions varied from never having reflected on existential support to considering it to be the most important aspect of nursing. Some nurses thought it was not included in their duties. It was unusual for nurses to reflect on existential support and they were unsure of what it involved. However in a separate study Strang, Strang, and Ternestedt [30] collected data from 141 nurses through questionnaires and found some nurses were able to identify patients who had spiritual needs and they placed importance on supporting patients through being a good listener, providing time, discussing the meaning of life, comfort, and serving as a link to other people.

A recent UK critical incident technique study of the spiritual needs of neuro-oncology patients illuminate that some of these patients have spiritual needs that could be met by nurses [23]. Although this was an exploratory study based on critical incidents from patients, it was indicative that nurses attempted to respond to their patients’ spiritual needs. The lack of further research in this area and the fact that to date the spiritual care of neuro-oncology patients has only been investigated in Sweden highlights the necessity of researching nurses caring for patients in the United Kingdom.

## Method

### Aims

The aim of this research was to begin to identify how neuro-surgical nurses in a UK Neurosurgical Unit manage the spiritual needs of neuro-oncology patients.

### Design

A qualitative approach based on Critical Incident Techniques (CIT) was used which involved collecting and analysing reports of behaviours in defined situations [31]. CIT was originally used by Flanagan [32] and has been used on numerous occasions in nursing research [23,32-35]. Flanagan [32] defines a critical incident as:

“any observable human activity which is sufficiently complete in itself to permit inferences and predictions to be made about the person performing the act.”

CIT was used in state of observation due to the practical constraints of using observation in the clinical setting. CIT has an advantage over highly structured interviews/ questionnaires which are too limited for this relatively unexplored area of nursing. CIT offers opportunities for obtaining descriptions of actual events and is therefore more concerned with what happens in the real world rather than an imagined world of how things should be [22,34,36]. CIT prompts participants to reflect upon nursing activities in the context of meanings and feelings concerning situations pertaining to nursing which they may otherwise be unable to articulate, if other methods are used [37]. Data can be obtained promptly with CIT as it is largely based on what participants do routinely [31]. A questionnaire format provided a quick and easy tool to collect the data and is considered to be most appropriate to CIT [31].

### Participants

The study’s sample comprised neurosurgical nurses who cared for patients with neuro-cancer patients to meet the aims of the study. Data were collected from nurses who had worked on the neurosurgical unit for patients with brain tumour at a large teaching hospital for a minimum of 6 months. The neurosurgical nurses worked in units where patients were admitted for biopsy and/or a crionotomy and debulking of their tumour since the onset of their illnesses. Patients’ diagnoses included grade II, III or IV glioma and anaplatic meningioma. Eighteen registered nurses who had worked on a 28 bedded neuro-surgical ward for at least 6 months were identified from a copy of the off duty and were sent letters of invitation and a copy of the questionnaire. Twelve completed questionnaires were returned. Although this was a low response the researchers felt that participants provided sufficient incidents for analysis. These respondents completed questionnaires related to nursing incidents of spiritual care consisting of the following:

1. Description of a nursing situation which led to the event or incident, when and how you as nurses recognised that your patients had spiritual needs; and when this occurred.

2. (a) What you thought that patients` spiritual needs were?

  (b) What led you to think this?

3. Description of what you did to try to help your patients and their families to meet their spiritual needs.

4. Description of the effect on the patients/family of what you did and explanation of that conclusion.

Prior to the administration of above questionnaire, the questions in the earlier version were piloted and refined to above statements to make them clear to illicit appropriate responses from participants. We administered the questionnaire following further consultations with research supervisors and internal reviewers who deemed it to be fit for the purpose of the research.

### Data analysis

We used a classification system based on a template analysis to analyse the data as per previous CITs [23,33,36,38]. We scrutinised the questionnaires to organise the data into meaningful segments under pre-determined headings. This provided an initial template for analysis which was subsequently revised to develop further sub-categories.

We constructed conceptual files for each identified category [39] following manual organisation of the data. We then typed all meaningful segments under each of these identified categories with appropriate headings. The meaningful segments were subjected to comparative analysis to ascertain similarities and differences to help us understand what kind of phenomena they reflected. The meaningful segments were subsequently converted to subcategories to reflect emerging themes.

The segments from the data were organised under the headings, ‘nurses awareness of their patients spiritual needs’; strategies to meet neuro-oncology patients’ spiritual needs’, ‘nurse reported relative spiritual needs,’ and ‘effective of nurse implemented strategies to meet relatives’ spiritual needs’. Having followed the above procedure we then reviewed the data again to capture the themes that emerged from the text. These were achieved by our meticulous review of the text to ensure that the themes emerged from the data. Some revisions were made to the themes following discussion among us as investigators to arrive at a final thematic presentation of the data as per findings section.

### Ethical considerations

Ethical approval was obtained from the former South Yorkshire Health Authority’s Research Ethics Committee where the research data were collected. Furthermore, the Research and Development Department approval was also obtained from the hospital NHS trust prior to commencing the research.

## Results and discussion

The results are discussed in the order of the statements in the CIT questionnaire in terms of the aims of the study (see Table 1).

**Table 1:** The process and outcome of responding to patients’ spiritual needs

**A. How nurses became aware of patients’ spiritual needs**	**B. The nature of patients’ reported concerns**	**C. The nurses’ action**	**D. The outcome of nurses’ intervention**
By encouraging patients to talk and listening to them	Being calm and happy as a feature of patient’s spirituality	Providing company/reassurance	Difficult to follow-up patients’ outcomes, for example, time factor, patient died,
Looking for clues about patients’ spirituality/religion	Expression of loneliness, anger	Providing explanation/practical support	Factors other than spirituality may contribute
Through recognition of patient’s emotions	Displays of emotions	Showing sensitivity	Nurses feel that the support they provide is of any assistance
Assuming spirituality equates with and religion	Talking about personal beliefs	Creating positive caring environment	Being sensitive and respectful
Admission of lack of awareness of spiritual needs	Overt expressions about God	Providing religious support/referral to chaplaincy	Feeling uncomfortable when dealing with patients’ spiritual needs because of lack of awareness
Role uncertainty in spiritual care	Asking for spiritual leader to visit	Providing support patients’ relatives, for example discussing end of life decisions; supporting spouse who felt lost and unbalanced	Do not believe spiritual care is a priority and consider it to be a burden on practice

### Nurses awareness of their patients spiritual needs

#### Talking and listening

There were variations in the extent to which nurses recognised patients’ spiritual needs and the importance they attached to spiritual care supporting previous findings [29]. Some of the descriptions of spiritual needs that nurses provided include:

‘Talked to patient’… ‘Tried to talk to the patient about his family and the life that he has had. Talk about the positive aspects of other patients with tumours (although very few)’… ‘Discussed making the most of ones time available.’

We tried to encourage the patient to talk about how she was feeling.’ ‘Talked to patient’… ‘conversations’ ‘Letting/allowing the patient and family to express their anger – and not being afraid to let them do this – gave them trust/faith in the nursing care.’

Others described spiritual needs in terms of:

‘He seemed so calm and happy when his wife visited. Despite her distress, which she expressed to me, she was very calm, positive and reassuring to him. So I think his spiritual needs were to have a regular contact with his wife, she visited’.

Nurses engagement with their patients in terms of talking and listening is seen as central to patients’ care and this offers opportunities to get a holistic understanding of their patients which includes spiritual needs [11,23,40-42]. However, Draper and McSherry [43] dispute this on the grounds that talking and listening to patients does not constitute spiritual care and much more sophistication is required to offer spiritual care. Others challenge such stance and argue that therapeutic communication executed effectively entails processes which go beyond social niceties in which a deeper level of connection with patients is possible [44]. In this regard, Johnstone Tayler [44] asserts that such communications enables nurses to understand the ‘inner person –the spirit component’ in patients and enact appropriate responses commensurate with spiritual support. In other words, talking and listening offers opportunities such as what to say to patients when they want to talk about their spirituality and spiritual concerns, provided nurses have been prepared professionally to deal with such spiritual issues.

#### Showing sensitivity to patients’ emotions

Some nurses provided examples of incidents in which they perceived that they responded to patients’ spiritual needs which included showing sensitivity to their emotions.

‘In this situation I felt the patient needed a lot of emotional support and encouragement to do things himself such as feed himself…Spiritual needs shown by the patient’s mood, i.e., tearful and feeling lonely’

When some nurses recounted about patients spiritual distress they identified that patients could be physically strong yet be unwell in the spiritual sense,

‘He was reasonably physically strong…However it was obvious that beneath a relatively calm exterior he was in great distress – I would say spiritual distress.’

Although some of them acknowledged that they knew little about what the spiritual needs of their patients they were still reported incidents in which they displayed sensitivity to patients’ emotions.

‘*He felt alone.. Patient was very aggressive and believed he had found God. He was asking us about our beliefs and asking for us to get someone to see him. He was also talking out loudly to God’.*

These nurses were sensitive to patients’ emotions which may well be manifestations of spiritual needs [45]. According to Rosseau [45] spiritual suffering is complex and nebulous and it is often difficult to assess, but acknowledges that it may manifest as physical or psychological problems and share features with depression such as states of despair, hopelessness and meaninglessness. However, alternatively some of the needs construed as spiritual are actually manifestations of psychological needs. For instance, emotional support could be identified as responses to psychological needs. We appreciate the overlap between spiritual and psychological needs as a human experience which is the sum of their biological, psychological and spiritual parts which are all interconnected. However, there is evidence to suggest there are differences between the psychological and spiritual aspects because there is an element to a person’s spiritual being that is independent of their psychology [23]. Spirituality is considered to be the essence of being and one that provides meaning and purpose to their existence and this does not come under the realms of psychology [46]. For example Swinton [9] asserts that a person’s spirituality can manifest itself through psychological processes but it also reaches beyond the psychological.

Since spiritual distress is difficult to assess nurses are likely to have varying degrees of success in meeting patients’ spiritual needs. However, in our study nurses provided incidents in which they were clearly sensitive to patients’ spiritual needs. Golberg [40] suggests that nurses carry out spiritual care at an unconscious level and that if nurses could be educated to be consciously aware of spiritual issues then patient care could be improved. However, others suggest many nurses were uncomfortable in dealing with patients’ spiritual needs [12,41]. Further work is needed to establish how nurses level of insight into spiritual issues affects the spiritual care they provide.

#### Religion and spirituality were viewed as interchangeable

Some nurses appeared to view religion and spirituality interchangeably:

‘They initially didn’t express a view on spirituality. After a while I looked in the admission file and noted that they were practising in their religion.’

‘It said he belonged to this religion on the admission documents. They didn’t express any other view. The patient and relatives didn’t say anything. The information was on the nursing notes.’

This aspect of the findings confirms the concern in the literature about the confusion over the term spirituality which is sometimes conflated with religion [4,7]. These nurses appeared to expect spiritual needs to be expressed in a very concrete way and to be literally reported by patients. However, nurses need to acknowledge that some patients regard their spiritual/religious beliefs as private matters and may appear reticent on such issues. Nurses therefore need to be sensitive and respectful to patients’ wishes [8]. Nurses need to develop an awareness to pick up cues about patients’ spiritual/religious needs and their manifestations, such as displays of anger and emotion.

#### Lacking experience in dealing with spiritual needs

Other nurses reported having no experience of dealing with the spiritual needs of neuro-oncology patients. Nurses may have no experience of spiritual needs as the neurosurgical setting is very intensive causing nurses to be less likely to identify patients’ spiritual needs. There has been some previous speculation in the literature that nurses in intensive ward settings are less likely to anticipate patients’ spiritual needs than nurses in palliative care [14]. However, some nurses indicated they were more aware of patients’ spiritual needs now on reflection than they had been at the time.

‘At this point I didn’t realise that this poor woman had spiritual needs’; ‘I could or should have’.

This aspect of the findings supports the proposition in nursing that critical reflective thinking brings about transformation [46] and in this instance it is clear that participants became aware of their patients’ spiritual needs as per Narayanasamy’s study [46] which offers insights into the many encounters which are described as spiritual in nature following his reflexive accounts of his research into spirituality in nursing.

#### Role uncertainty in spiritual care of cancer patients

Although nurses acknowledged that on reflection, patients may have spiritual needs, the lack of awareness in practice may be due to time constraints imposed by the acute setting. According to Koenig [24] the lack of time appears to be a major factor in contemporary health care practice. No nurses in the current study questioned whether providing spiritual support was a nursing role although one nurse indicated it wasn’t a role that nurses in the acute setting would get involved with. One nurse reflected:

‘I’m sorry I have no experience of this. I think our area is too acute. From (personal) experience I think may be spiritual needs are important at the point of diagnosis or later when the acute phase is past. May be it would be better to target the oncology nurses.’

It would be interesting to know how nurses with this view would define spirituality and spiritual needs. Nurses may be identifying spiritual needs of patients and addressing these needs without recognising them as spiritual needs due to the confusion over the term spirituality because there is the lack of consensus on what is meant by spirituality [1]. In this regard, Schenrich [7] suggests that spirituality should be substituted by terms such as ‘meanings and values of life’ to overcome the existing ambiguity about spirituality among health care professionals. Such definitions may be more familiar to nurses and hence they are more likely to discuss meaningfully about cancer impacting upon patient’s sense of identity and values of life. However, clearer definitions of what spiritual and religious care are, emerging, for example, NHS Scotland's Guidelines for Spiritual and Religious Care in the NHS in Scotland [47] offers a clear description of the two terms:

**Religious care** is given in the context of the shared religious beliefs, values, liturgies and lifestyle of a faith community.

**Spiritual care** is usually given in a one-to-one relationship, is completely person centred and makes no assumptions about personal conviction or life orientation. Spiritual care is not necessarily religious. Religious care, at its best, should always be spiritual.

Cassidy and Davies [48] acknowledge that such a helpful distinction may alleviate the misappropriation of spirituality to fit religious interventions by health professionals.

The issue of nurses’ role uncertainty in spiritual care of cancer patients has been found previously [49], and there is evidence that nurses caring for neuro-oncology patients do not believe spiritual care is part of their role [29]. Nurses may not want responsibility for spiritual care, not because they don’t perceive it as important but because spirituality is not seen as a priority in the healthcare system within which they work. Spirituality can end up being perceived as an added burden within this highly pressured environment due to financial, political, and economic constraints within the system [50]. However, Timmons and Narayanasamy [51] suggest that the UK NHS is a secular organisation and therefore tries to be impartial on matters of spirituality although they also observe that the sacred and the secular may co-exist in reality.

In summary, we have sketched out descriptions of nurses awareness of their spiritual needs and spirituality in the context of the incidents they had provided. However we acknowledge that they reacted differently in their spiritual care performance and awareness and these are probably indicative of the varying levels of preparedness, experience, beliefs and values positions related to spirituality and spiritual care. There is evidence in the literature that some nurses are more sensitive to patients’ spiritual needs and prepared to invest their time in addressing such needs [13]. Further evidence suggests that nurses who had educational preparation in spiritual care are most likely to respond to their patients’ spiritual needs and implement nursing interventions to meet such needs [2]. Other evidence indicates that nurses who are religious are mostly likely to respond to patients’ spirituality [13,19].

### Strategies to meet patients’ spiritual needs

Common nursing strategies to meet spiritual needs reported by nurses were flexibility with hospital policies, communication link to family, privacy, religious support, supportiveness, company, and reassurance. See above **Table**1: **C**. **The Nurses**’ **Action** for findings from this aspect of the study.

The finding that nurses identify religious support as a strategy nurses can use is consistent with the results of other studies [11,29,30,44]. However, a preference for cancer patients to receive spiritual care from the chaplain rather than nurses was also found by Highfield [52]. Whether patients choose to gain spiritual support from the chaplain because this is what they truly prefer or because they don’t recognise spiritual care as a nursing role is a question to be answered in future research. It is important for nurses to realise that faith and belief can be sources of spiritual strength for religious people [11,30]. The nurses in these two studies did not report recognising belief outside of the religious context. However it may also be due to the fact that the nurses participating in Strang, et al’s., [30] study were from palliative, oncology, neurology, and psychiatric settings as well as neurosurgery. Neurosurgical nurses may not be as in tune with some aspects of spirituality as nurses working in other settings. The finding that nurses recognise providing a communication link between patients and family is important for neuro-oncology patients as supported by previous research [30]. Family support has been identified previously as an effective coping strategy by neuro-oncology patients [17]. Nurses can ensure family relationships are maintained through ensuring telephone messages are relayed and relaxing visiting times, if necessary.

Many of the strategies identified by nurses including providing explanations, sensitivity, sitting with and talking to patient, allowing expression of emotions, comfort, empathy, and providing time and listening, all resonate with the concept of ‘presencing’ as described previously [40,42]. This finding supports the notion that this is an important component of spiritual care. There are differing opinions within the nursing literature regarding the use of talking and listening as strategies to meet patients’ spiritual needs. Draper and McSherry [43] argue that although talking, listening, and sitting with the patient are important aspects of holistic care they do not come under the remit of spiritual care. However, as discussed earlier, others dispute this stance because nurse-patient communication offers opportunities for connection at a deeper level which bring about therapeutic benefits, including spiritual benefits such as peace, hope, strength and comfort to patients [1,11,44].

### Relatives’ Spiritual needs and strategies to meet them

Nurses recognised that relatives of patients had spiritual needs and that there were several strategies they used to support them. Two categories were established during analysis of the nurse data: relative spiritual needs and strategies to meet these needs. These categories and the further subcategories that were identified are illustrated in Tables 2 and 3.

**Table 2:** Nurse reported relative’s spiritual needs

**Relative spiritual needs**	**Illustrative examples from the nurse questionnaire data**
End of life decisions	*(1) ‘I feel they [the family] felt I was not exploring other options i.e. – not writing a living will. Although they expressed they would support her decision I felt they didn’t really want her not to be ventilated again.’*
Feeling of being lost and unbalanced	*(2) ‘The wife of one man…. she had spoken to several doctors, and in fact any new face she encountered, asking the same questions and receiving the same answers again and again. As yet another new face she repeated her questions to me. The other friends and relatives with her appeared embarrassed for her. To me she looked lost and totally unbalanced. The answers she was receiving was not what she actually wanted to know or hear’*
	*(3) ‘At this point I didn’t realise that this poor woman had spiritual needs’*

**Table 3:** Nurse implemented strategies to meet relative spiritual needs

**Nurse implemented strategies to meet relative spiritual needs**	**Illustrative examples from the nurse questionnaire data**
Encourage exploration of meaning of life	*(1) ‘I spoke not of diagnosis and prognosis. But of their lives, loves and achievements together. Somehow leading her to use what they had built between them in the past into becoming something to strengthen her through into the future without him.’*
	*(2) ‘Someone would come along who would put it all into place for her and that is what I had done.’*
Time and privacy to talk	*(3) ‘I took her and a friend into a room alone and began to talk to her.’*

Nurses in our study made a point of highlighting how they helped patients’ relatives with their spiritual needs. Narayanasamy and Owen [41] found in their study that some nurses were willing to fully immerse themselves in all aspects of their patients’ care, including care of their relatives. They showed deep understanding and sensitivity when addressing patients’ and their relatives’ spiritual needs. Other studies suggest that patients’ relatives and carers valued spiritual care support from nurses and other health care professionals [3,53].

### Effectiveness of nurse interventions to meet patients spiritual needs

As previously indicated, nurses in this study reported that they were aware of their patients’ and relative’s spiritual needs. These included the need for talking and listening to patients’ spiritual and religious beliefs, emotional needs, showing sensitivity to patients’ emotions, responding to religious needs; and responding to relatives’ spiritual needs included: supporting them with end of life decisions, supporting them when feeling being lost and unbalanced, encouraging exploration of meaning of life and providing space, time and privacy to talk. The nurses’ actions such as providing company and reassurance, providing explanation and practical support, showing sensitivity, creating positive caring environment and support for their relatives were indicative of effectiveness of their interventions. Nurses gave specific statement of outcomes to support their claims about such effectiveness. These included patients’ states such as ‘appearing calm and happy’ when nurses encouraged them to talk and acted as sounding board. However, they were also concerns that their interventions could be better. Such accounts reflected uncertainty about the effectiveness of their support. Some of these accounts included:

‘The space of time that I care for patients with malignant brain tumours is very short. They are admitted to our unit high on both steroids and hope that the surgery will cure them.’

This may be because the nurse participants were all staff nurses working in an acute neurosurgical setting where neuro-oncology patients spend a short period of time and intervention effectiveness follow-up is difficult. This is further illustrated as follows:

‘unfortunately I have not chased up what happened.’

‘..and the outcome is hard to know the outcome as they died and one cannot know their true feelings,...’

‘It is difficult to assess the effect it had, as there were clearly other issues affecting the patient ie. diagnosis/prognosis. At times when we arranged for her to go home she had to come straight back into hospital when her condition deteriorated. It was a challenge balancing her physical, psychological and spiritual needs as meeting one often meant neglecting another. Ultimately the patient went home and I did not see her again so am not sure if our actions had a positive impact or not.’

The acute nature of the neurosurgical setting is problematic for implementing and evaluating spiritual care. This is in contrast to the oncology setting where length of hospitalisation is long, so providing increased opportunities for nurses and patients to build a rapport and share spiritual and existential reflections [14]. There was also the sense that nurses didn’t always feel that the support they provided for patients was adequate:

‘I’didn’t feel that any of what I did was of any real assistance to the patient, but I do feel that it helped give the family the support that they required – to lose a husbad and father whilst he is still alive must be one of the most horrendous experiences.’

‘I could or should have offered some kind of support earlier’

Strang et al. [17] found that nurses don’t always feel prepared to offer spiritual support to neuro-oncology patients supporting previous research findings. Ross [12] draws from pedagogical research evidence to advocate that nurses’ knowledge and competence in spiritual care could be developed through spiritual care education. A recent study conducted by O’Shea et al. [54] found that the ASSET Model [46] was an effective framework for teaching spiritual care education and it improved nurses’ knowledge and understanding of spiritual care needs in patients and families. Likewise, Van Leewen and Cusveller [55] offer useful nursing competencies for spiritual care derived pedagogical research but Ross [12] suggests that these require further testing for effectiveness.

### Methodological problems and limitations

As an alternative to CIT, collecting the data through interviews may have enhanced the quality and detail of the data collected. However, all nurse data were included and were treated in the same way. Many of the nurse incidents did not fit the specified criteria probably due to the difficulty of knowing the outcomes of care in an acute environment.

Although CIT appeared to have many advantages at the design stage of the study it may not have been the most appropriate tool to use as it required nurses to recall incidents pertaining to patients’ care and spirituality. With time lapse, memories of significant incidents may fade and valuable information may be lost. This may explain why many of the reports were quite general. The reliance of CIT on the memories and perceptions of participants has been noted as a limitation previously [34,36]. Martin and Mitchell [34] reported that if the incident is too sensitive for participants, they may withhold information, which maybe the case in our study in some instances. An interview format may be more successful as the researcher could guide the participant to stay focused on the specific incident. Cox, et al. [37] use of CIT with cancer patients may have been successful because they used interviews. However, such interviews are open to potential bias because of the temptations to ask leading questions to retrieve the responses researchers wanted.

It is likely that nurses in the present study had difficulty describing a complete critical incident due to the acute nature of the environment in which observation and follow up of care is difficult and complex. If nurses provide spiritual care but are unable to follow it up, then this may present them with an ethical dilemma. McSherry and Ross [8] raise ethical questions about what happens to patients who are discharged with unresolved spiritual issues or whose spiritual needs are at a crucial stage in being resolved, but truncated because nurses are unable to sustain spiritual support. Nurses caring for neuro-oncology patients report that although they appreciate the need for engaging in supportive conversations about existential issues, time constraints may prevent them from sustaining conversations involving profound reflections [17]. They are fearful of initiating something such as deeper conversations about existential issues often pertaining to patients’ spirituality. These nurses fear that they could not sustain or finish such conversations. This raises the question of whether nurses in the acute environment should be addressing spiritual issues if they will be unable to see this through due to time constraints and additional priorities such as responding to the physical effects of cancer on patients. On the other hand it is unethical to leave a patient in spiritual distress simply because nurses don’t have time. More nursing resources need to be provided or other professionals must receive training in spiritual care in order to take responsibility for this role in the neurosurgical setting.

## Conclusions

This research illuminates that the critical incidents from nurses indicate that some patients with brain tumours have spiritual needs and nurses do respond to these needs. The findings are indicative that nurses concur what the spiritual needs of neuro-oncology patients are and how these can be met. There is a strong indication from this research that some nurses have clear perceptions about their patients’ spiritual needs. Some nurses identified strategies that could be used by nurses to assist neuro-oncology patients with their spiritual needs. Although further research is required to establish the differences between spiritual and psychological needs, we maintain the distinctness of spirituality in terms of meaning and purpose in one’s life. Any disruption to meaning and purpose impacts upon the holistic well-being of a person leading to spiritual distress. For example, this may manifest as anxiety (fear and uncertainty), depression (states of hopelessness and despair). In this context it would be reasonable to speculate that spiritual support which helps patients to regain their meaning and purpose would alleviate their spiritual distress, hence amelioration of manifestations of anxiety or depression. However, we do acknowledge the role of psychiatry and psychotherapeutics in the treatment of anxiety and depression as one of the methods of intervention in dealing with acute psychological crises. However, Aldridge [56] suggests that critical junctures such as diagnosis of cancer evoke spiritual crisis requiring spiritual support to alleviate distress in patients.

Although we could not get a conclusive picture of how effectiveness nursing interventions related to spiritual care was ‘presencing’ [40,42] may be a major strategy nurses can use to support patients’ spiritually. It goes without overstating that it is clearly an important part of nurses’ role to provide humane, caring and compassionate care to their very ill neurosurgical patient, however, some scholars [40,42] suggest that ‘presencing’, the act of being with patients is a form of spiritual support. This is particularly necessary even when nurses are lost for appropriate words or ‘what say say when patients want to talk about spirituality?’ According to Johnstone Taylor [57], a therapeutic presence with gestures of unconditional regard and warmth to patients will go a long way to support patients who are very ill.

Besides being there or presencing, nurses can provide spiritual support for neuro-oncology patients that are flexible with hospital policies, encouraging family relationships, providing privacy and appropriate religious support.

The finding that nurses believe there is no place for spiritual care in the neurosurgical setting is indicative that there may be a need for further education of nurses on the nature of spirituality and spiritual needs of neuro-oncology patients. The fact that some nurses may only be practising spiritual care on an unconscious level hints at the need for further education of nurses in spiritual care. McSherry and Jamieson [2] claim that there is anecdotal evidence that nurses require additional guidance and support on matters related to spiritual and religious needs of patients. Nurses who have received education in spiritual care feel more able to provide spiritual care to patients [54]. In future research it would be interesting to look at the extent of spiritual care education received by neurosurgical nurses in the UK and compare it to their attitudes to spiritual care of neuro-oncology patients. However, we appreciate that it requires years of experience to horn these sophisticated human skills that are essential for spiritual care. However, there is evidence to suggest that nurses could be trained to provide spiritual care. For example, the ASSET Model [46] has been used successfully as a theoretical framework for spiritual care education in evaluation studies. Although there is the ethical risk that nurses who are not well trained in spiritual care may cause more harm than good when they attempt to provide spiritual care, we suggest that nurses should, at the least, receive preparation in terms of professional development to identify patients’ spiritual needs and refer such patients to hospital chaplains or spiritual advisers who are well placed to provide spiritual support for patients. However, if nurses are well trained in matters of spirituality and spiritual care then they are most likely to address patients’ spiritual needs when these occur as other professionals such as chaplains and spiritual advisers do not normally work round the clock as nurses do. However, this statement does not undermine health care practices where chaplains or spiritual advisers are readily available all the time.

Further research is required to explore how nurses can support neuro-oncology patients with their spiritual needs and whether this should in fact be a nursing role. This research does however serve its purpose as a preliminary study providing evidence that spiritual care of neuro-oncology patients is important within the neurosurgical setting.

## Competing interest

The authors declare that they have no competing interests.

## Authors’ contributions

**AVN** was responsible for the study conception and design, data analysis and manuscript drafting. **AN** was responsible for supervising the study, critical review of the analysis and manuscript. **VP** was responsible for a critical review of the analysis and manuscript. All authors read and approved the final manuscript.

## Pre-publication history

The pre-publication history for this paper can be accessed here:

http://www.biomedcentral.com/1472-6955/12/2/prepub
